# Multilocus sequence analysis of *Anaplasma phagocytophilum* reveals three distinct lineages with different host ranges in clinically ill French cattle

**DOI:** 10.1186/s13567-014-0114-7

**Published:** 2014-12-09

**Authors:** Amélie Chastagner, Thibaud Dugat, Gwenaël Vourc’h, Hélène Verheyden, Loïc Legrand, Véronique Bachy, Luc Chabanne, Guy Joncour, Renaud Maillard, Henri-Jean Boulouis, Nadia Haddad, Xavier Bailly, Agnès Leblond

**Affiliations:** INRA, UR346 Epidémiologie Animale, F-63122 Saint Genès Champanelle, France; Université Paris-Est, Ecole Nationale Vétérinaire d’Alfort, UMR BIPAR, 23 avenue du Général de Gaulle, 94706 Maisons-Alfort, France; INRA, CEFS, UR035, 24 chemin de Borde Rouge - Auzeville, CS 52627, F-31326 Castanet Tolosan, France; LABÉO - Frank Duncombe, Unite Risques Microbiens (U2RM), Normandie Universite, EA 4655 Caen, Normandy France; Laboratoire Vétérinaire Départemental du Rhône, Campus vétérinaire VetAgro Sup, 1 avenue Bourgelat, 69280 Marcy l’Etoile, France; Université de Lyon, VetAgro Sup, Jeune Equipe Hémopathogènes Vectorisés, F-69280 Marcy l’Etoile, France; Groupe Vétérinaire de Callac, 26 rue du Cleumeur, 22160 Callac, France; Ecole Nationale Vétérinaire de Toulouse, Unité pathologie des ruminants, 23 Chemin des Capelles, 31076 Toulouse, France; Département Hippique, VetAgroSup, F-69280 Marcy L’Etoile, France

## Abstract

Molecular epidemiology represents a powerful approach to elucidate the complex epidemiological cycles of multi-host pathogens, such as *Anaplasma phagocytophilum. A. phagocytophilum* is a tick-borne bacterium that affects a wide range of wild and domesticated animals. Here, we characterized its genetic diversity in populations of French cattle; we then compared the observed genotypes with those found in horses, dogs, and roe deer to determine whether genotypes of *A. phagocytophilum* are shared among different hosts. We sampled 120 domesticated animals (104 cattle, 13 horses, and 3 dogs) and 40 wild animals (roe deer) and used multilocus sequence analysis on nine loci (*ankA, msp4, groESL, typA, pled, gyrA, recG, polA,* and an intergenic region) to characterize the genotypes of *A. phagocytophilum* present. Phylogenic analysis revealed three genetic clusters of bacterial variants in domesticated animals. The two principal clusters included 98% of the bacterial genotypes found in cattle, which were only distantly related to those in roe deer. One cluster comprised only cattle genotypes, while the second contained genotypes from cattle, horses, and dogs. The third contained all roe deer genotypes and three cattle genotypes. Geographical factors could not explain this clustering pattern. These results suggest that roe deer do not contribute to the spread of *A. phagocytophilum* in cattle in France. Further studies should explore if these different clusters are associated with differing disease severity in domesticated hosts. Additionally, it remains to be seen if the three clusters of *A. phagocytophilum* genotypes in cattle correspond to distinct epidemiological cycles, potentially involving different reservoir hosts.

**Electronic supplementary material:**

The online version of this article (doi:10.1186/s13567-014-0114-7) contains supplementary material, which is available to authorized users.

## Introduction

Molecular characterization of the genetic diversity present within pathogen species provides valuable information that can be used to develop appropriate monitoring or control measures [1]. Understanding a pathogen’s intraspecific genotypic diversity can aid in diagnosis, specifically by allowing the detection of different variants. Moreover, studies examining the host specificity of individual pathogen genotypes can aid in planning control measures for pathogens that circulate among multiple host species. Such measures could involve managing host diversity in order to reduce disease risk [2]. For example, in the UK, research on the genetics of the host-pathogen interaction between badgers and the tuberculosis-causing bacterium *Mycobacterium bovis* has helped to improve strategies for limiting tuberculosis in cattle [3]. Studies of genetic diversity can also clarify the epidemiology of vector-borne pathogens that, as a result of the low host specificity of their vector(s), circulate among multiple vertebrate hosts. This is the case for ticks, which may have different hosts during different developmental stages [4]. Transmission patterns of tick-borne pathogens can thus be quite complex and depend on a pathogen’s ability to infect different host species. Initially, the *Ixodes*-borne pathogen *Borrelia burgdorferi* was thought to be a single bacterial species [5]. However, subsequent molecular characterization revealed the presence of multiple variants, each with a specific host range, a result that has highlighted the complexity of the epidemiological cycles of this tick-pathogen association [6].

*Anaplasma phagocytophilum* is a pathogen that raises similar questions. This tick-borne bacterium is the etiologic agent of granulocytic anaplasmosis, an emerging disease that affects a wide range of mammals [7,8]. In cattle, it causes reduced milk yield, abortion, and immunosuppression, which facilitates secondary infections [9,10]. At present, disease control is limited to treating symptomatic cases with antibiotics; little preventive action is taken, since both the source(s) of the pathogen and the environmental conditions that favor the infection of cattle remain poorly documented.

In wild fauna, *A. phagocytophilum* is highly prevalent and persistent in cervids, which are consequently considered to be a reservoir for this bacterium. In areas where *A. phagocytophilum* is endemic, its prevalence is commonly higher than 70% in both roe deer *(Capreolus capreolus)* and red deer *(Cervus elaphus)* populations [11,12]. However, the strains carried by roe deer appear to be different from those carried by domesticated animals [13,14]. Indeed, molecular studies have shown that different variants of *A. phagocytophilum* circulate among different hosts, which suggests that each bacterial strain could be associated with a different epidemiological cycle [15-17]. A thorough genetic characterization of *A. phagocytophilum* could help determine which hosts contribute to each cycle and, more importantly, which hosts promote the spread of the bacterium in cattle.

Various molecular techniques have been used to characterize the genetic diversity of *A. phagocytophilum*. These include restriction fragment length polymorphism [18], pulsed-field gel electrophoresis [19], and multiple-locus variable number tandem repeat analysis [20]. Such techniques have yielded limited information due to either a lack of resolution (the two former techniques) or an excess of variability (the latter technique). The direct detection of polymorphism in DNA sequences has proven to be a more versatile method. Thus far, the characterization of *A. phagocytophilum* has been based on the sequencing of one or a few loci. The most frequently used markers are *groESL*, *ankA*, *msp4*, and 16S rRNA. All these markers except 16S rRNA have revealed high levels of diversity, but phylogenies based on single genes have yielded inconsistent results [13,21,22]. The use of seven or more markers can help resolve such phylogenetic incongruence. Different markers provide information that can be used to identify different genotypic groups, also called clonal complexes, that belong to a common lineage and that are closely related to each other [23]. Two recent studies have used this multilocus sequence typing approach to explore the intraspecific diversity of *A. phagocytophilum* in different host species. One examined dogs, sheep, roe deer, and humans in Europe [24], while the other investigated roe deer and red deer in Poland [25]. Using analyses of seven housekeeping genes and four loci, respectively, these studies have provided robust support for well-defined groups of strains.

In this study, we used multilocus sequence analysis to describe the genetic diversity of the *A. phagocytophilum* strains that infect domestic mammals in France. We employed nine loci, including three commonly studied genes (*groESL*, *ankA*, and *msp4*), and six new markers*.* Using this approach, we investigated the genetic diversity of *A. phagocytophilum* strains that were responsible for clinical cases of bovine granulocytic anaplasmosis throughout the country. Our goal was to identify the bacterial variants circulating within the cattle population. We then attempted to determine additional host species that could be involved in the circulation of bovine strains. To do this, we compared the genotypes associated with the bovine clinical cases to the genotypes circulating in horses, dogs, and roe deer in France. We discuss our results in the context of other studies that have investigated *A. phagocytophilum*’s genetic diversity in different reservoirs.

## Materials and methods

### Samples

All animal samples were collected in France between 2011 and 2013. We obtained samples from clinically ill domesticated animals via the network of veterinary clinics belonging to the French national association SNGTV (Société Nationale des Groupements Techniques Vétérinaires) and governmental veterinary laboratories (GVL). More precisely, blood or DNA extracts that had been prepared from different tissues (Additional file 1) were obtained from 104 cattle, 13 horses, and 3 dogs. All these animals had tested positive for granulocytic anaplasmosis after they expressed clinical symptoms compatible with the disease. During the same period, blood samples were taken from wild and captive-bred roe deer sampled as a result of deer management efforts and a population monitoring study. In total, 40 roe deer representing 3 regions were included in our study (Additional file 1).

To prepare the DNA extracts, whole blood samples were stabilized with EDTA, and 200 μL of each sample was used in the DNA extraction procedure. DNA extraction took place in a silica column, and we employed a Nucleospin Blood QuickPure Kit (Macherey-Nagel, Düren, Germany) in accordance with the manufacturer’s instructions. Some DNA extracts arrived directly from the GVLs; all had been prepared in the same way, although the brand of column-based DNA extraction kits varied among laboratories and according to biological material.

The presence of *A. phagocytophilum* was assessed in each DNA sample with real-time PCR. For this, we used a protocol adapted from Courtney et al. [26] that targeted the *msp2* and *p44* genes. The reaction mix included 5 μL of DNA extract, 10 μL SsoFast Supermix (Biorad, Hercules, California), 2 μL of each primer (10 μM ApMSPf and ApMSP2r), and 0.5 μL of the Taqman probe ApMSP2p-HEX (10 μM), in a total volume of 20 μL.

### Selection of loci

To characterize the genetic diversity of the bacteria causing the infections, we chose nine loci that are evenly distributed across the genome of *A. phagocytophilum.* Among these were *ankA*, *msp4*, and *groESL,* which have frequently been used to characterize the diversity of *A. phagocytophilum.* In addition, six other loci were specifically selected for this analysis. The biological function of the selected genes was not the principal criterion in locus selection, as had been initially proposed for MLST schemes by Maiden et al. [27]. Indeed, Konstantinidis et al. suggested that this strategy was rather conservative [28]. They showed that randomly chosen sets of orthologous genes always contain phylogenetic information that correlates with phylogenetic information at the whole genome scale. Furthermore, this pattern holds despite the fact that the strength of the correlation varies greatly among sets of genes, probably because of the species-specific recombination rate.

A single *A. phagocytophilum* genome was available at the beginning of this study (*A. phagocytophilum* strain HZ GenBank: NC_007797.1). Consequently, the choice of the six new loci could not be based on either the phylogenetic information they contained or the conservation of genes among *A. phagocytophilum* genomes. We thus selected markers by comparing the HZ genome with the genomes of *Anaplasma marginale* (strains St. Maries GenBank: NC_004842.2 & Florida GenBank: NC_012026.1) and the genome of the *Anaplasma centrale* Israel strain (GenBank: NC*_*013532*.*1). In particular, we looked for markers that were shared among *Anaplasma* strains and identified six targets: three house-keeping genes (*gyrA*, *polA*, and *recG*), a gene coding for a GTP-binding protein (*typA*), a gene encoding a response regulator linked to intracellular infection (pleD, [29]), and an intergenic spacer located in a region of high synteny (*APH_1099-APH_1100*).

### Locus-specific amplification of DNA

Because of the low bacterial load within the blood samples, we first increased the amount of DNA so that more would be available for the amplification of the molecular markers. Multiple displacement amplification (MDA) was performed on positive samples using the Illustra GenomiPhi V2 DNA Amplification Kit (GE Healthcare Life Sciences, Buckinghamshire, UK) in accordance with the manufacturer’s recommendations. The DNA solution obtained was diluted in 80 μL of purified water.

Each locus was then amplified using nested PCR. In the first PCR, each reaction used 5 μL of pre-amplified DNA in a total reaction volume of 50 μL. The reaction mix included 2 units of Taq polymerase (Qiagen, Venlo, Netherlands), 4 μL of each primer (at a concentration of 10 μM), 4 μL of dNTPs (at a concentration of 25 mM), 10 μL of Q solution (Qiagen), and 2 μL of 25-mM MgCl_2_. Prior to sample amplification, tests were performed to optimize the annealing temperatures. The PCR program began with an initial denaturation step of 3 min at 95 °C. This was followed by three cycles that consisted of a denaturation step of 1 min at 95 °C, an annealing step of 2 min at the temperature described for the published primers for *groEL* [30] and other primer pairs (Additional file 2), and an extension step of 90 s at 72 °C. The program continued with 40 cycles that differed from the first three by having a denaturation step of 1 min at 88 °C and ended with an extension step of 10 min at 72 °C. The nested PCR was performed using 5 μL of the product of the first PCR in a total volume of 50 μL, with the same mix and cycling conditions as in the primary amplification.

### DNA sequencing and alignment

Nucleotide sequences were obtained from PCR products using Sanger sequencing (Beckman Coulter Genomic, Essex, UK). When PCR amplification with the external primers was successful in recovering an amount of PCR product that was sufficient for sequencing, we sequenced this longer fragment. However, when it was unsuccessful, the shorter fragment obtained from the nested PCR was used. For DNA fragments longer than 1000 bp, sequencing was performed on both strands. Base calls were checked manually in ChromasLite version 2.1, and IUPAC codes were used to indicate ambiguous states. DNA sequences were aligned using the “*Clustal Omega*” algorithm in SeaView version 4. The sequences analyzed in this study are available in Genbank under the accession numbers KJ832158-KJ833031.

### Single-locus polymorphism analyses

Loci were amplified with differing degrees of success because bacterial DNA concentrations varied across samples. Additionally, the primers showed differing sensitivity to individual sequences. For this reason, each locus was initially examined independently in the polymorphism analyses. All samples were retained even if they had not yielded sequences for all target loci. We wanted to ensure that a similar amount of phylogenetic information could be obtained from the different sequences at each locus. We therefore excluded from further analyses any sequences that were shorter than 70% of the expected length. We also excluded sequences containing ambiguous base-calls.

To estimate polymorphism at each locus, DnaSP 5.10 software was used to calculate haplotype diversity (Hd, the number of variable nucleotide sites) and nucleotide diversity (π, the average number of nucleotide differences per site between two sequences). We also investigated the potential impact of recombination on our alignments by calculating minimum recombination values (Rm).

### Phylogenic tree building

Unless otherwise indicated, the following analyses were carried out with the packages “ape”, “ade4”, and “fpc” in R v2.15.1 [31].

For each locus, we first used the alignment constructed for the polymorphism analyses to build a DNA distance matrix. We then used a bio++ algorithm to integrate the parameters of the best model of evolution for that alignment (selected with the Akaike Criterion (AIC) value calculated using the *phymltest* function in “ape”). Using this DNA distance matrix, a phylogenic tree was built with the *bionj* function in “ape”. When possible, similar analyses were performed using homologous data obtained from GenBank (data not shown).

Then, we restricted the alignment of each locus (except that of *ankA*) to samples for which at least five out of our eight loci had been successfully sequenced. The goal was to create a supertree. As before, the AIC of each locus was calculated from the restricted dataset in order to select the best model of sequence evolution. Then, the alignments of the eight loci were concatenated into a single alignment. As samples included in the supertree analysis could lack up to three loci, missing locus sequences were represented by gaps in the concatenated alignment. A supertree was built from the concatenated alignment with MrBayes 3.2 software, using a Bayesian method of construction that has been developed to highlight the information provided by the different markers while optimizing tree structure [32]. This method has been shown to be robust even when more than 50% of the data are missing [33].

The data were partitioned in MrBayes, and the respective best model of evolution was applied to each partition. Three runs of 500 000 generations were performed using a Metropolis-coupled Markov chain Monte Carlo (MCMCMC) approach based on six chains. Trees were sampled from the main chain every 500 generations. Assuming that convergence had not been reached, the first 25% of sampled trees were removed. Then, using the remaining trees, a supertree was generated from the 25% of trees with the highest posterior probabilities among the three runs. The robustness of the phylogenetic relationships among genotypes was evaluated using the Bayesian posterior probability of the branches.

### Phylogenetic analyses

The presence of clusters in the phylogenic supertree was evaluated using the Ward’s classification approach [34] based on the supertree distances; this was calculated using the *hclust* function in R. The number of clusters was determined using the minimum sum of squared errors and confirmed with the functions *pamk* and *kmeans* in the “fpc” package. Each sample present in the supertree was assigned to one cluster. The clusters from the supertree were mapped onto the *ankA* phylogenic tree in order to compare the two phylogenic structures.

We then wanted to explore if DNA polymorphism at each locus was linked with either host origin or the clusters identified in the supertree. To do this, a discriminant analysis was performed on the principal coordinate analysis of the DNA distance matrices using the *dudi.pco* function in the “ade4” package.

## Results

### Polymorphism analyses

All the results of the polymorphism analyses are summarized in Table 1.

**Table 1:** **Genetic diversity measured for all loci with DnaSP**

	**APH 1099-1100**	***Msp4***	***RecG***	***PolA***	***GyrA***	***TypA***	***GroESL***	***PleD***	***AnkA***	***AnkA*** **I**	***AnkA*** **II**	***AnkA*** **III**	***AnkA*** **IV**	**Mean**
**Nb indiv**	98	136	88	79	54	114	100	63	101	34	6	16	45	
**Length bp**	435	390	351	333	837	462	630	597	654	661	753	710	702	
**Nb site poly**	19	38	12	21	20	27	35	27	352	47	22	36	74	
**% site poly**	3.37	9.74	3.41	6.30	2.39	5.84	5.55	4.52	53.82	7.11	2.92	5.07	10,54	6.575
**Nb haplo**	9	30	17	22	24	33	36	10	64	22	4	9	31	
**Haplo Div (Hd)**	0.565	0.875	0.807	0.838	0.93	0.86	0.923	0.661	0.983	0.961	0.8	0.908	0.958	0.827
**Nucleo div (π)**	0.0084	0.0237	0.0047	0.0106	0.0043	0.0087	0.01156	0.0168	0.2083	0.0176	0.0123	0.0076	0.0185	0.0178
**Rm**	1	7	1	5	6	7	7	2	NA	8	0	1	12	

Nb indiv: number of samples analyzed by loci; Length bp: length of the alignment in bp; Nb site poly and % site poly: number and proportion of polymorphic sites in the alignment; Nb haplo: number of haplotypes; Hd: haplotype diversity; π: nucleotide diversity; and Rm: number of estimated recombination events.

The sequence lengths of the alignments obtained for the different loci ranged from 333 to 837 bp, excluding gapped and missing sites (837 bp for *gyrA*, 630 bp for *groESL*, 654 bp for *ankA*, 597 bp for *pleD*, 462 bp for *typA*, 390 bp for *msp4*, 351 bp for *recG*, and 333 bp for *polA*). Haplotype diversity ranged from 0.565 to 0.983, with an average diversity of 0.827. The loci *msp4*, *gyrA*, *groESL*, and *ankA* demonstrated above-average haplotype diversity, while the haplotype diversity of *APH_1099-1100*, *recG*, and *pleD* was below average.

We treated *ankA* separately from the other loci because it is composed of four very divergent regions. Its overall average nucleotide diversity was 0.2. However, closer examination of this locus revealed that, while the start and the end regions of its sequences were obviously homologous, more than 50% of sites were polymorphic. As a result, it was difficult to estimate nucleotide diversity and the number of recombination events for the locus as a whole. Consequently, we chose to examine the sequences of the four clusters (named I to IV) separately.

When all loci were taken into account (including *ankA* I-IV), intralocus nucleotide diversity ranged from 4.7 × 10^−3^ to 2.3 × 10^−2^, with an average of 1.8 × 10^−2^ nucleotides per site. The values for *msp4*, *groESL*, *pleD*, and *ankA* I-IV were higher than the mean, while *recG* and *gyrA* had the lowest diversity, with 4.7 × 10^−3^ and 4.3 × 10^−3^ nucleotide differences, respectively, per site between pairs of sequences.

### Phylogenic trees and analyses

The different phylogenic trees built from single gene alignments were not directly comparable to each other because of both conflicting phylogenetic signals and missing data. Indeed, we were able to sequence all nine loci for only 11 samples. However, in each phylogenic tree, the genotypes obtained from roe deer clustered in one or two branches that were only distantly related to most other genotypes (Additional file 3). This clustering of roe deer genotypes was also supported by the discriminant analysis of all the loci (Additional file 4).

**Figure 1:**
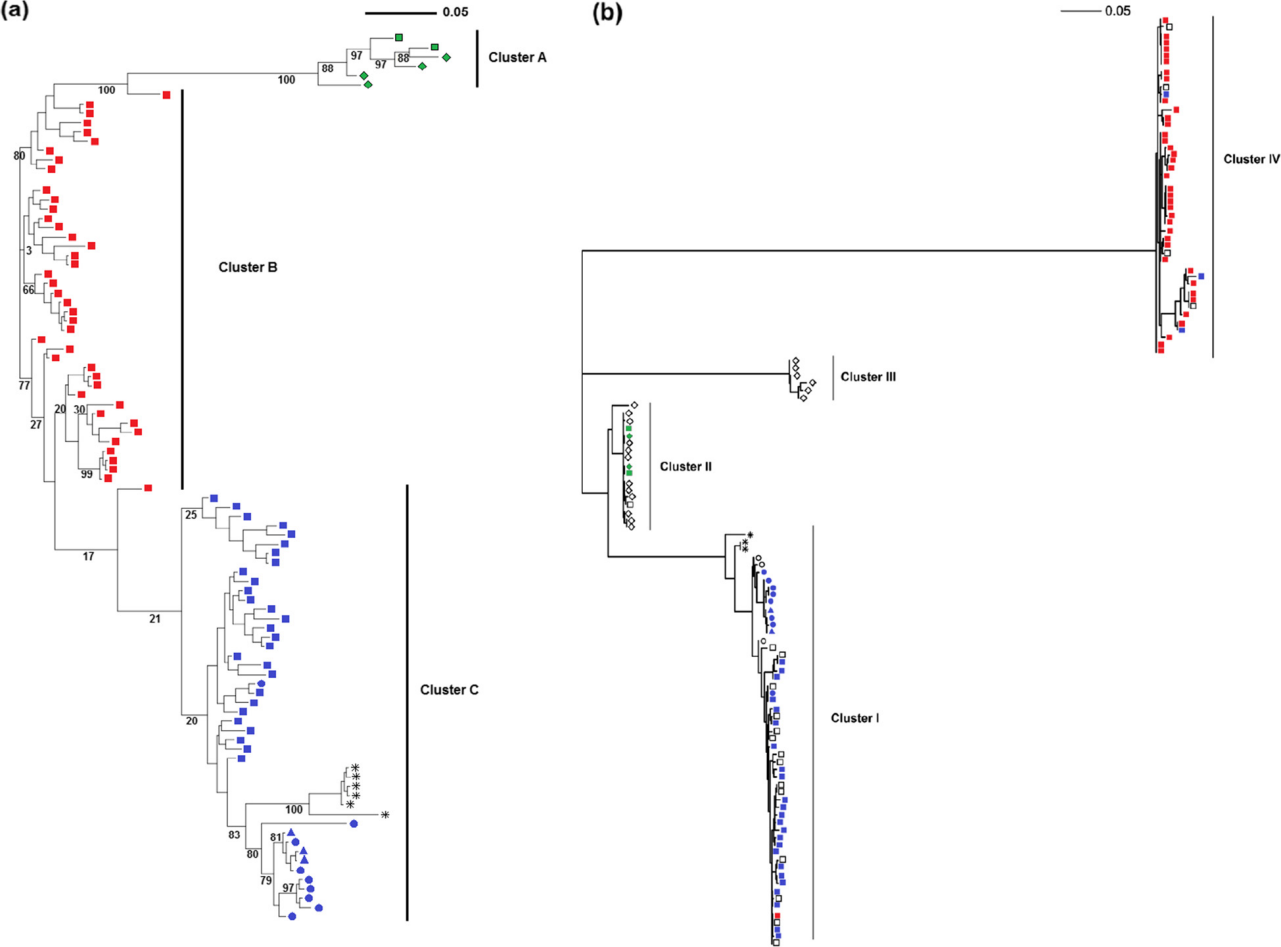
**Unrooted supertree (a) & unrooted** ***ankA*** **tree (b).** Each tree was built using a maximum likelihood approach. The supertree was built from parameters optimized by MCMCMC with MrBayes, and the *ankA* phylogeny was built using parameters optimized with *phymltest* in R. Each sample is identified by a symbol representing its host animal: □ for cattle, ○ for horses, ◊ for roe deer, and ∆ for dogs; the different colors indicate the supertree cluster to which the sample was assigned.

**Figure 2:**
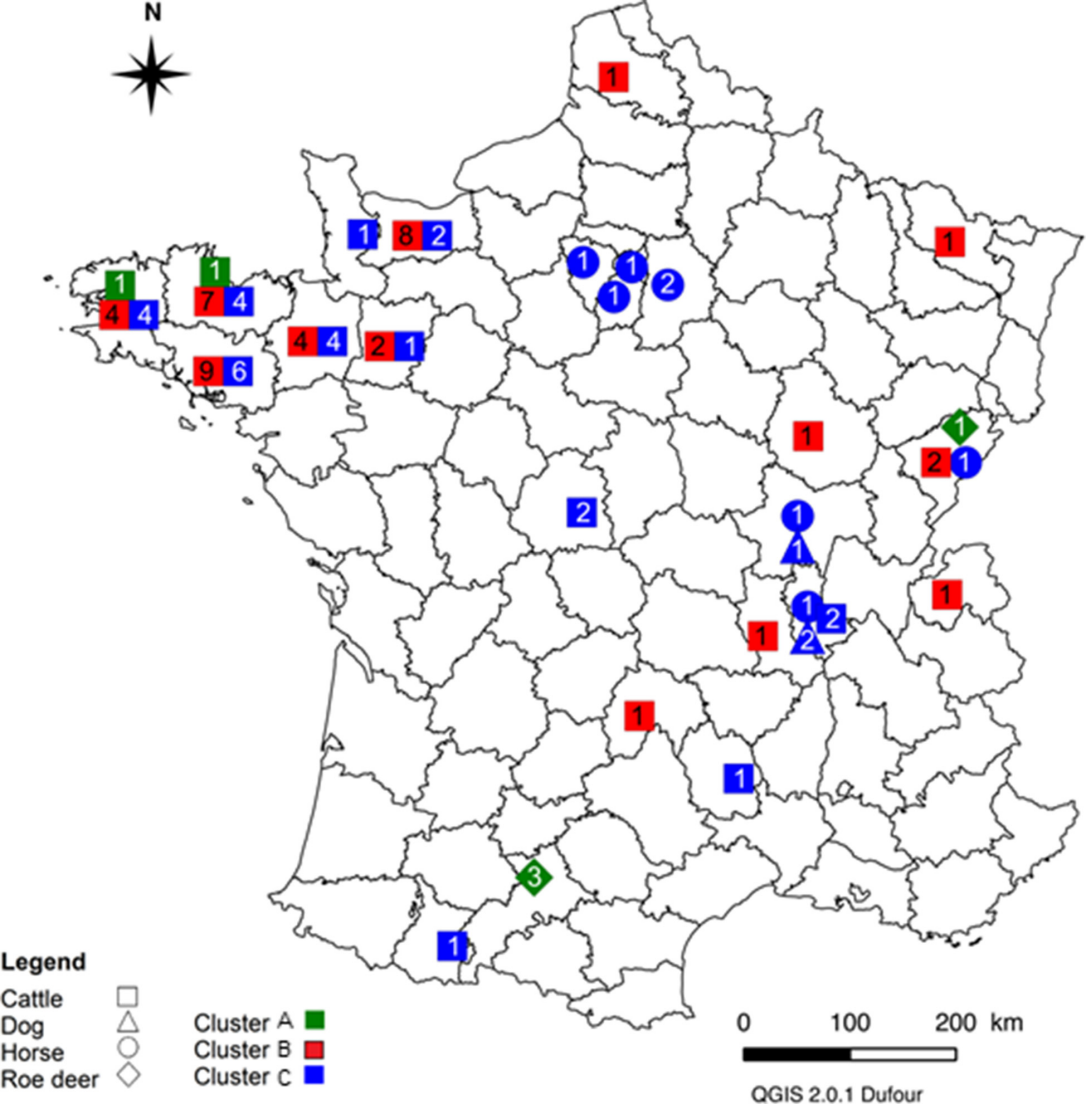
**Geographical distribution of supertree clusters.** Samples are grouped into their French region (“departement”) of origin; the shape of each symbol corresponds to the host animal from which the samples were recovered, while the color corresponds to the supertree cluster to which samples were assigned. The number of isolates represented by each symbol is provided within the symbol.

For each locus (with the exception of *ankA*), we generally found that measurements of diversity were relatively homogenous, while most branching patterns were heterogeneous. With this in mind, we decided to build a supertree, based on all loci except *ankA*, to obtain more insight into the phylogenetic relationships among *A. phagocytophilum* genotypes. The supertree was thus based on a sub-sample of 93 bacterial genotypes for which at least five loci had been characterized. This dataset included individual genotypes obtained from 72 cattle, 9 horses, 4 roe deer, and 3 dogs (Figure 1). Within the tree, the cluster analysis revealed the presence of three genetic groups. Cluster A included all the roe deer and two cattle genotypes (BR-BO-46 and BR-BO-08); cluster B was composed exclusively of cattle genotypes; and cluster C was composed of the horse, dog, and remaining cattle genotypes. In general, the clusters identified in the supertree were not observed in the single-locus phylogenies, with the exception of the *pleD* phylogeny. No obvious geographical patterns were observed in the clustering of the different genotypes (Figure 2). However, we could not test the correlation between geographical and phylogenetic distance because of the low number of individual samples per host species, as well as the lack of precise information about sample location.

The *ankA* phylogeny generated here was structured in four clusters (designated I, II, III, and IV), as has been previously described [13]. For 95% of the genotypes, the pattern of clustering in the *ankA* phylogeny was highly similar to that of the supertree. In general, the genotypes of supertree cluster A grouped in *ankA* cluster II, the genotypes of supertree cluster B grouped in *ankA* cluster IV, and the genotypes of supertree cluster C grouped in *ankA* cluster I. Only four genotypes, all isolated from cattle, were found in a different cluster in the supertree than in the *ankA* phylogeny (BR-BO-09, RA-BO-03, BR-BO-14, and NO-BO-26). However, although the phylogenic relationships were topologically similar between the supertree and the *ankA* tree, the phylogenic distances between the clusters were very different. For example, we observed fewer than 0.05 substitutions per site between clusters B and C in the supertree; in contrast, the inferred divergence between clusters I and IV in the *ankA* tree (which contained the same genotypes) was nearly one substitution per site.

In the past, *ankA* cluster II had been reported to contain *A. phagocytophilum* genotypes isolated exclusively from ticks, roe deer, and red deer. However, our study found three cattle genotypes (BR-BO-46, BR-BO-08 and BR-BO-63) in this cluster. To our knowledge, this is a novel finding. Our results agree with previously published studies in showing that *ankA* cluster III only includes variants found in roe deer: six genotypes from roe deer grouped in this cluster [13]. Unfortunately, these samples could not be included in the supertree because the minimum criterion of having at least five sequenced loci was not met. The discriminant analysis of *ankA* revealed an association between host species and phylogenetic distance between genotypes, which was not found in the phylogenies of any of our other markers.

## Discussion

High-resolution genetic data are important to decipher potentially genotype-dependent components of epidemiological cycles—such as sources and chains of transmission—and the spatial distributions of diseases. In this study, we characterized the diversity of *A. phagocytophilum* genotypes found in cattle using a combination of three commonly used markers and six new ones. The new markers were included in order to improve the degree of resolution of our genotyping efforts. We constructed a supertree based on eight of those markers and compared its structure with that of a tree based on the *ankA* gene, which is linked to infection. In both trees, bovine *A. phagocytophilum* genotypes grouped into three major clusters. This finding strongly supports the existence of major genetic lineages in populations of *A. phagocytophilum*, a pattern that is probably due to host adaptation.

Our results support the value of these markers in studying the genetic diversity of *A. phagocytophilum*. Indeed, the degree of polymorphism found in our study allowed us to characterize multilocus genotypes that corresponded to most of the *ankA* groups described in the literature. The average pairwise divergence among genotypes (i.e., π) in our samples was lower for the new loci (*typA*, *gyrA*, *pleD*, *recG*, *polA*, and the intergenic spacer *APH_1099-APH_1100*) than it was for *groESL* and *msp4*. It was also much lower than the *ankA* values, suggesting that the new genes we chose contain relevant phylogenic information. Haplotype diversity, which represents the probability of finding different alleles in a pair of samples, was similar across most loci; it had an average value of 0.8. This result also suggests that the added loci contributed to our ability to discriminate among genotypes of *A. phagocytophilum*.

Because of the unusual pattern of genetic diversity at the *ankA* locus, it was used to construct a separate tree, an approach that has been applied in other studies*.* The *ankA* locus is a widely used marker that enables large-scale genetic delineation among genotypes of *A. phagocytophilum*. Previous studies of *ankA* diversity have found that four highly divergent groups—designated groups I, II, III, and IV—exist within *A. phagocytophilum* [13]. The topology of our *ankA* phylogeny reflected this same grouping. However, the specific evolutionary constraints of *ankA* have resulted in a degree of genetic divergence among groups that is too high to allow detailed inference about phylogenic relationships among *A. phagocytophilum* lineages (e.g., up to one substitution per site has been inferred between clusters I and IV). Likewise, within-group *ankA* diversity is too low to provide an informative degree of resolution.

As expected from previous studies [35], the other single-gene phylogenetic analyses generated different topologies. However, regardless of the gene under consideration, alleles isolated from roe deer always grouped in one or two clusters that were distantly related to all other genotypes. There are several potential explanations for the phylogenetic incongruencies we observed among the single-tree phylogenies. First, they may reflect a lack of resolution resulting from the small number of informative sites per locus [23]. This could explain, for example, the presence of polytomies in the phylogenetic tree based on *typA*. Another potential explanation is the presence of recombination events. Indeed, when we calculated the minimum recombination values, we found evidence of recombination events. Such events have the potential to bias the inference of phylogenetic relationships. This problem has already been reported in a phylogenetic study based on *msp4* and *ankA* markers [36,37]. Finally, the incongruencies we observed could also be the result of some individuals being co-infected by multiple strains [38]. In such cases, the phylogenetic locations of loci obtained from the same host individual would be expected to vary across the single-locus trees because the sequences would have come from different, co-infecting strains. In our data, the presence of ambiguous sites in some chromatograms suggests that multiple strains may have been present in some samples. This problem appeared to rarely affect the cattle samples but may have had an influence on around 30% of the roe deer samples. We thus excluded all samples with ambiguous sites at least one locus to avoid the risk of incongruence between loci.

These three factors may have increased inconsistency in the single-gene phylogenies. Because of this, we employed another approach that uses several markers—and thus the phylogenetic information they contain—to generate robust genotype clusters [23]. Indeed, the supertree approach, here based on a Bayesian method of construction, was designed to highlight the common information revealed by markers while optimizing tree structure [32]. Moreover, supertree construction is commonly used to deal with the problem of missing data [39]. This is another recurring problem in *A. phagocytophilum* studies, since the rate of successful amplification in naturally infected samples can be lower than 30% [17]. However, the dataset used to build our supertree contained less missing data and thus allowed us to obtain an informative clustering pattern [33,40].

In our supertree, we identified three divergence-based clusters that almost perfectly matched the topologies of *ankA* groups I, II, and IV [13]. Unfortunately, we were not able to include the roe deer genotypes assigned to *ankA* cluster III in our supertree. Their inclusion would have allowed us to further investigate the distribution of *ankA* alleles among *A. phagocytophilum* lineages and represents an interesting future research goal. From a phylogenetic point of view, the relationships among strains were more clearly resolved in the supertree than in the *ankA* phylogeny. This was the case even though most of the inner branches of the supertree were not as robustly supported, which was to be expected given the issues discussed above [33]. However, the various divergence-based clusters do not represent monophyletic groups in the supertree. The discovery of a related outgroup or the acquisition of more phylogenetic information could give further insight into the evolution of lineages within *A. phagocytophilum*. Indeed, a recent genomic study suggested that up to 50 markers may be required to achieve the resolution that is necessary to investigate the phylogenetic relationships among conspecific bacterial genotypes [23].

The similar genetic clusters formed in both the supertree and the *ankA* tree further support the idea that major genetic lineages exist within populations of *A. phagocytophilum*. The three clusters in our supertree appear to each be linked to a different host species community: cluster B was composed exclusively of cattle genotypes; cluster C was composed of genotypes found in cattle, horses, and dogs; and cluster A was composed of the all roe deer genotypes and two genotypes found in cattle. The strain divergence we observed may be explained by three factors. First, there may be epidemiological differences (e.g., different host reservoirs or vector species) leading to different epidemiological cycles [17,41,42]. Second, hosts may differ in their susceptibility, which could lead to the emergence of bacterial genotypes with different levels of virulence [43]. Third, different lineages may have emerged or evolved in different geographical areas [44]. Among these three possibilities, the first—that strain epidemiology may differ—seems to be the best supported by our results.

The hypothesis that different strains have different epidemiological cycles is supported by the observation that the topologies of the single-gene trees based on *pleD* and *ankA* showed the same clustering patterns as the supertree. These two genes are known to be involved in the infection process. The former is one of three response regulators of *pleC*, a gene predicted to encode a sensor kinase. Both *pleC* and *pleD* are synchronously upregulated during the exponential growth stage of *A. phagocytophilum* and downregulated prior to extracellular release in human patients [29]. The latter encodes an effector protein secreted by the type IV secretion system that has been implicated in several infection processes. In particular, AnkA facilitates intracellular infection by activating the Abl-1 signaling pathway [45] and by manipulating host cell processes in its interactions with the *Anaplasma* translocated substrate 1 (Ats-1) protein [46]. Furthermore, *ankA* interacts with other gene regulatory regions of host chromatin to downregulate expression of key host defense genes like CYBB (gp91^phox^) [47]. From a phylogenetic point of view, genes involved in the infection process are highly relevant because selective pressures applied to those genes could promote host specialization. Indeed, phylogenetic analyses of *ankA* have identified clusters that are strongly linked to specific host communities. For example, clusters II and III are composed predominantly of strains isolated from roe and red deer [13], while the recently described cluster V includes only rodent strains [37]. However, it may also be that the evolution of *A. phagocytophilum* as a species is driven by two opposing selective pressures, one towards specialization in a given host species and the other favoring a broader host range [48,49]. The close association between the *ankA*-based genetic clusters and the host communities indicates that there may be a barrier to cross-species transmission, which could arise from the host-specific nature of infection pathways. This relationship also suggests that the optimized fitness of a strain in one host species comes at the cost of lower fitness when the strain infects other species. An alternative evolutionary strategy could involve infecting a large number of susceptible species in order to persist longer in host communities. This strategy could prove especially useful if the species composition of the community varies over time. Assuming that different bacterial variants have the opportunity to come in contact with different host species, the ability of a given *A. phagocytophilum* genotype to infect different host species would be key to its persistence in the local community. Selection may also favor an intermediate strategy—the maintenance of diverse variants with overlapping host ranges. This is what we found here, with three clusters of genotypes that each contained cattle-infecting strains. Other studies have shown that it is possible for populations of *A. phagocytophilum* to have co-existing epidemiological cycles, with different genotypes infecting different sets of hosts [12,17,41]. In this case, the divergence among strains would be linked to adaptation to different sets of hosts rather than to a single species [49].

The second hypothesis is that differences in host susceptibility have led to the emergence of divergent *A. phagocytophilum* genotypes that demonstrate different degrees of virulence. If genetic variation underlies the virulence of *A. phagocytophilum* genotypes, this fact may explain the variety of symptoms that have been described in cattle, including edema, fever, abortion, as well as acute or chronic symptoms [10]. Mathematical models support the idea that pathogen strains that differ in their degree of virulence can emerge within a single host species [43]. However, this outcome requires that a large degree of variation exist in individual susceptibility within that species (as a result of vaccination, for example). In contrast, the emergence of variable virulence is easier to observe in multihost systems [50]. In this case, a shift in the pathogen’s host range would likely involve a concomitant evolutionary shift in virulence. It can be hypothesized that limited transmission between the respective reservoirs of different genotypes might eventually lead to variation in virulence. Consistent with this hypothesis is the observation that different *A. phagocytophilum* genotypes isolated from different host species showed different degrees of virulence when they were used to experimentally infect lambs [51] and mice [52]. In the present study, we found that the phylogenetic clustering of genotype groups in the supertree mirrored the clustering observed in the single-gene trees that were based on infection-related genes. This pattern strongly suggests that the evolution of *A. phagocytophilum* genotypes is influenced by selective pressures on genes that regulate infection. The genetic variation that we observed in these genes may well be linked to variation in virulence, and it is possible that strains from different genotype clusters provoked different symptoms in infected animals. However, since we lacked sufficiently detailed information about the individual symptoms of each host animal, we were not able to test this hypothesis.

The third hypothesis states that genetically divergent strains of *A. phagocytophilum* may have evolved in different geographic regions. However, this hypothesis is not supported by our data, as we did not observe clear, broad-scale heterogeneity in the spatial distribution of the genotypes (Figure 2). It is nonetheless important to note that spatial (environmental or geographical) factors could structure pathogen populations. This could occur through drift or through changes in the host community involved in the transmission cycle. For example, there exists an European *A. phagocytophilum* genotype only observed in the Camargue and in Sardinia. This suggests that the environmental conditions of the Mediterranean region favor the dispersion of this genotype [36,53]. Similarly, it has been hypothesized that host distribution and other environmental constraints influence the local distribution of *A. phagocytophilum* lineages in Sicily [54]. In this study, however, we did not observe any clear clustering of genotype groups that could be attributed to geographical boundaries. Divergence among clusters is thus more likely explained by a difference in host communities rather than by geographical factors.

Consequently, of the three hypotheses discussed above, this study found support only for the first: that the genotype clustering we observed stems from strains having different epidemiological cycles. Therefore, we will further discuss the three clusters of *A. phagocytophilum* genotypes found in cattle, working from the assumption that they are the result of three independent epidemiological cycles.

In the supertree, clusters A, B, and C all included genotypes isolated from cattle. Within the latter, a subgroup of genotypes corresponded to those found in *ankA* cluster I. This subgroup contained genotypes associated with cattle as well as genotypes associated with dogs and horses. Although we had relatively few equine and canine samples, these results are consistent with those previously reported for *ankA* [13]. Furthermore, a recent study also showed that this cluster of *ankA* genotypes includes not only strains that infect horses and dogs, but also those that infect other hosts, such as humans, wild boar (*Sus scrofa*), hedgehogs *(Erinaceus europaeus)*, and red deer [24]. Since *A. phagocytophilum* has high levels of prevalence in red deer and hedgehogs (approximately 60% and 80% of individuals are infected, respectively [14,55-57]), further quantitative surveys are needed to assess whether these hosts play a role in the spread of this *A. phagocytophilum* strain in cattle.

In our analysis, supertree genotype cluster B, corresponding to *ankA* cluster IV, contained only strains isolated from cattle, although previous studies have reported related genotypes in sheep and, rarely, in roe and red deer [13,37]. The seemingly low level of prevalence of this bacterial lineage in wild mammals raises questions about the role of these animals in the spread of this genotype. Livestock are not generally thought of as reservoir species for *A. phagocytophilum* because they only sporadically exhibit clinical symptoms of anaplasmosis. However, a serological survey in cattle herds indicated that pathogen prevalence may be high—it varied from 31 to 77% at different times of the year [58]. Furthermore, longitudinal surveys in cattle herds have suggested that the immunity acquired following exposure to *A. phagocytophilum* does not suffice to prevent subsequent infection but is sufficient to prevent clinical signs of disease. This could result in a high proportion of asymptomatic carriers [59]. Interestingly, experimental studies have shown that *A. phagocytophilum* can persist in “reservoir tissues” in sheep; while they may be persistent carriers, PCR analyses of their blood will fail to detect infection [60]. It is possible that the bacteria present in “reservoir tissues” periodically circulate in the blood and infect feeding ticks. This possibility underscores the need for further experimental studies on *A. phagocytophilum* infection in cattle: if the same process occurs in cattle, the bovine population could act as a reservoir.

Finally, a few bovine genotypes were found in cluster A of the supertree, which corresponds to *ankA* cluster II. In the multigene phylogeny, this group was relatively distantly related to the others and was dominated by genotypes from roe deer. From an epidemiological point of view, it is likely that roe deer contribute to the spread of *A. phagocytophilum*, if only because the pathogen is highly prevalent in roe deer populations (up to 70% of infection detected by PCR, 11, 12). From a genetic point of view, a phylogenetic analysis of *ankA* indicated that the genotypes carried by roe deer and a few red deer were distantly related to the genotypes carried by other host species [13]. Similarly, previous studies using *groESL* have indicated that the strains carried by roe deer are different from the strains found in other hosts [14]. Finally, a recent study showed that both MLST genotypes and *ankA* sequences supported the existence of a roe deer-associated group of *A. phagocytophilum* genotypes [24]. After the publication of more than 800 *ankA* sequences and 380 MLST-characterized sequences in the literature, we report here, for the first time, that strains isolated from cattle are present within this cluster. Our results suggest that, however rare it may be, transmission can occur between cattle and roe deer and may be due to a spillover effect.

In conclusion, we used multilocus sequencing to describe the genetic diversity of *A. phagocytophilum* strains causing clinical illness in domesticated animals in France. In doing so, we discovered the presence of three groups of genotypes. These groups show a cross-country distribution and are probably associated with different host communities, an observation that suggests that *A. phagocytophilum* is propagated through different epidemiological cycles that involve different reservoir and host species. In order to gain further insights into the population dynamics of this bacterial pathogen, future epidemiological studies are needed. These studies should be organized on a subregional scale and focus on different potential reservoirs, domesticated hosts, and lineages of *A. phagocytophilum*. Such a sampling scheme would be challenging to carry out, so epidemiological modeling is also required to determine the number of infected individuals that should be sampled within a potential reservoir species to adequately describe pathogen flow. In addition, the detection and characterization of asymptomatic cases in cattle—whether these cases are due to variation in pathogen virulence or to differences in symptom manifestation in different hosts—represents an important research path to explore. In particular, the existence of healthy carriers that contribute to the maintenance and spread of virulent strains could have serious implications for efforts aimed at limiting bovine granulocytic anaplasmosis.

## Additional files

Additional file 1:
**Description of the host and geographical origin of the samples and the classification of the** ***ankA*** **and supertree clusters to which the samples were assigned.** The table contains information on the samples used in the study. a: “-” indicates ambiguous or too short sequences; “NA” indicates unsuccessful amplification. b: “-” indicates individuals not included in the supertree because less than five loci were characterized. Additional file 2:
**Primers used in and information about the nested PCRs.** The table contains information on the primers used in the study. * developed by Lotrič-Furlan [30]. Additional file 3:
**Unrooted trees based on the sequences of the following loci: a)** ***groEL***; **b)** ***msp4***; **c)** ***gyrA*** **; d)** ***pleD*** **; e)** ***polA*** **; f)** ***recG*** **; g)** ***typA*** **; and h) the intergenic region C** ***trA-*** ***APH_1100.*** Each tree was built using a maximum likelihood approach; parameter optimization was performed with *phymltest* in R. The colors indicate the supertree cluster to which the samples were assigned: green for the cluster A, red for the cluster B, and blue for the cluster C. Additional file 4:
**Discriminant analysis by host based on the principal coordinates analyses of the following loci: a)** ***msp4*** **; b)** ***polA*** **; c)** ***typA*** **; d)** ***groEL*** **; e)** ***recG*** **; f)** ***CtrA***- ***APH_1100*** **; g)** ***gyrA*** **; and h)** ***pleD.*** The graphs show the results of the discriminant analysis conducted on the principal coordinates analysis of each locus.
