# Supplementary material for: Multilocus sequence analysis of Anaplasma phagocytophilum reveals three distinct lineages with different host ranges in clinically ill French cattle
Source: Vet Res. 2014 Dec 9;45:114. doi: 10.1186/s13567-014-0114-7 (PMC4334609; doi:10.1186/s13567-014-0114-7)
Supplement: Additional file 1: — Description of the host and geographical origin of the samples and the classification of the ankA and supertree clusters to which the samples were assigned. The table contains information on the samples used in the study. a: “-” indicates ambiguous or too short sequences; “NA” indicates unsuccessful amplification. b: “-” indicates individuals not included in the supertree because less than five loci were characterized. [file 13567_2014_114_MOESM1_ESM.pdf]

Sequence_id	Species	Tissue	French region (« département »)	*ankA* cluster[a]	Supertree cluster[b]
AU-BO-01	CATTLE	blood	Cantal (15)	4	B
BL-HO-01	HORSE	blood	BELGIUM	1	-
BL-HO-02	HORSE	blood	BELGIUM	-	C
BO-BO-01	CATTLE	blood	Côte-d'Or (21)	4	B
BR-BO-02	CATTLE	blood	Idre (Idre (36))	4	-
BR-BO-03	CATTLE	blood	Côtes-d'Armor (22)	1	C
BR-BO-04	CATTLE	blood	Côtes-d'Armor (22)	4	B
BR-BO-05	CATTLE	blood	Tarn-et-Garonne (82)	1	-
BR-BO-06	CATTLE	blood	Côtes-d'Armor (22)	1	C
BR-BO-07	CATTLE	blood	Ille-et-Vilaine (35)	1	C
BR-BO-08	CATTLE	blood	Finistère (29)	2	A
BR-BO-09	CATTLE	blood	Côtes-d'Armor (22)	4	C
BR-BO-10	CATTLE	blood	Ille-et-Vilaine (35)	-	C
BR-BO-11	CATTLE	blood	Morbihan (56)	1	C
BR-BO-12	CATTLE	blood	Aveyron (12)	1	-
BR-BO-13	CATTLE	blood	Morbihan (56)	NA	-
BR-BO-14	CATTLE	blood	Lozère (48)	4	C
BR-BO-15	CATTLE	blood	Finistère (29)	4	B
BR-BO-16	CATTLE	blood	Côtes-d'Armor (22)	4	B
BR-BO-17	CATTLE	blood	Morbihan (56)	4	B
BR-BO-19	CATTLE	blood	Côtes-d'Armor (22)	4	-
BR-BO-20	CATTLE	blood	Finistère (29)	4	B
BR-BO-21	CATTLE	blood	Finistère (29)	1	C
BR-BO-22	CATTLE	blood	Finistère (29)	1	C
BR-BO-23	CATTLE	blood	Morbihan (56)	4	B
BR-BO-24	CATTLE	blood	Morbihan (56)	4	B
BR-BO-25	CATTLE	blood	Morbihan (56)	4	B
BR-BO-26	CATTLE	blood	Morbihan (56)	1	C
BR-BO-27	CATTLE	blood	Morbihan (56)	1	C
BR-BO-28	CATTLE	blood	Morbihan (56)	1	C
BR-BO-29	CATTLE	blood	Finistère (29)	1	C
BR-BO-30	CATTLE	blood	Ille-et-Vilaine (35)	1	C
BR-BO-31	CATTLE	blood	Ille-et-Vilaine (35)	4	B
BR-BO-32	CATTLE	blood	Ille-et-Vilaine (35)	4	B
BR-BO-33	CATTLE	blood	Ille-et-Vilaine (35)	4	B
BR-BO-34	CATTLE	blood	Ille-et-Vilaine (35)	4	B
BR-BO-35	CATTLE	blood	Côtes-d'Armor (22)	4	B
BR-BO-36	CATTLE	blood	Côtes-d'Armor (22)	1	C
BR-BO-37	CATTLE	blood	Côtes-d'Armor (22)	1	-
BR-BO-38	CATTLE	blood	Morbihan (56)	4	B
BR-BO-39	CATTLE	blood	Côtes-d'Armor (22)	1	-
BR-BO-40	CATTLE	blood	Morbihan (56)	1	C
BR-BO-41	CATTLE	blood	Morbihan (56)	4	B
BR-BO-42	CATTLE	blood	Côtes-d'Armor (22)	4	B

BR-BO-43	CATTLE	blood	Côtes-d’Armor (22)	4	B
BR-BO-44	CATTLE	blood	Morbihan (56)	4	B
BR-BO-45	CATTLE	blood	Côtes-d’Armor (22)	4	B
BR-BO-46	CATTLE	blood	Côtes-d’Armor (22)	2	A
BR-BO-47	CATTLE	blood	Finistère (29)	-	B
BR-BO-48	CATTLE	blood	Finistère (29)	1	C
BR-BO-49	CATTLE	blood	Mayenne (53)	4	-
BR-BO-50	CATTLE	blood	Finistère (29)	NA	-
BR-BO-51	CATTLE	blood	Morbihan (56)	4	B
BR-BO-52	CATTLE	blood	Finistère (29)	1	-
BR-BO-54	CATTLE	blood	Finistère (29)	NA	B
BR-BO-55	CATTLE	blood	Côtes-d’Armor (22)	1	-
BR-BO-56	CATTLE	blood	Morbihan (56)	4	B
BR-BO-58	CATTLE	blood	Morbihan (56)	1	C
BR-BO-59	CATTLE	blood	Ille-et-Vilaine (35)	1	C
BR-BO-60	CATTLE	blood	Morbihan (56)	1	-
BR-BO-62	CATTLE	blood	Ille-et-Vilaine (35)	4	-
BR-BO-63	CATTLE	blood	Côtes-d’Armor (22)	2	-
BR-BO-64	CATTLE	blood	Finistère (29)	1	-
CE-BO-01	CATTLE	blood	Idre (36)	1	C
CE-BO-02	CATTLE	blood	Idre (36)	NA	-
CE-BO-03	CATTLE	blood	Idre (36)	1	C
CRL-12	ROE DEER	blood	Doubs (25)	NA	-
CRL-19	ROE DEER	blood	Doubs (25)	NA	-
CRL-22	ROE DEER	blood	Doubs (25)	3	-
CRL-3	ROE DEER	blood	Doubs (25)	NA	A
CRL-7	ROE DEER	blood	Doubs (25)	NA	-
FC-BO-01	CATTLE	blood	Doubs (25)	4	B
FC-BO-02	CATTLE	blood	Doubs (25)	4	B
IF-HO-02	HORSE	blood	Seine-et-Marne (77)	1	-
IF-HO-04	HORSE	blood	Seine-et-Marne (77)	1	C
IF-HO-05	HORSE	blood	Seine-et-Marne (77)	1	-
IF-HO-08	HORSE	blood	Essone (91)	1	C
IF-HO-09	HORSE	blood	Seine-et-Marne (77)	NA	-
IF-HO-10	HORSE	blood	Val-de-Marne (94)	1	C
IF-HO-11	HORSE	blood	Yvelines (78)	1	C
LI-BO-33	CATTLE	blood	Haute-Vienne (87)	NA	-
LI-BO-55	CATTLE	blood	Haute-Vienne (87)	1	-
LI-BO-80	CATTLE	blood	Haute-Vienne (87)	NA	-
LO-BO-04	CATTLE	blood	Moselle (57)	-	B
MP-BO-01	CATTLE	blood	Hautes-Pyrénées (65)	1	C
MP-BO-02	CATTLE	blood	Lot (46)	NA	-
MP-CH-02	ROE DEER	blood	Haute-Garonne (31)	2	-
MP-CH-05	ROE DEER	blood	Haute-Garonne (31)	2	-
MP-CH-06	ROE DEER	blood	Haute-Garonne (31)	3	-

MP-CH-08	ROE DEER	blood	Haute-Garonne (31)	-	-
MP-CH-10	ROE DEER	blood	Haute-Garonne (31)	3	-
MP-CH-11	ROE DEER	blood	Haute-Garonne (31)	-	-
MP-CH-12	ROE DEER	blood	Haute-Garonne (31)	3	-
MP-CH-14	ROE DEER	blood	Haute-Garonne (31)	3	-
MP-CH-15	ROE DEER	blood	Haute-Garonne (31)	-	-
MP-CH-16	ROE DEER	blood	Haute-Garonne (31)	2	-
MP-CH-17	ROE DEER	blood	Haute-Garonne (31)	2	-
MP-CH-19	ROE DEER	blood	Haute-Garonne (31)	3	-
MP-CH-24	ROE DEER	blood	Haute-Garonne (31)	2	-
MP-CH-25	ROE DEER	blood	Haute-Garonne (31)	2	-
MP-CH-26	ROE DEER	blood	Haute-Garonne (31)	2	-
MP-CH-29	ROE DEER	blood	Haute-Garonne (31)	2	-
MP-CH-32	ROE DEER	blood	Haute-Garonne (31)	2	-
MP-CH-33	ROE DEER	blood	Haute-Garonne (31)	2	-
MP-CH-34	ROE DEER	blood	Haute-Garonne (31)	-	-
MP-CH-35	ROE DEER	blood	Haute-Garonne (31)	-	-
MP-CH-39	ROE DEER	blood	Haute-Garonne (31)	2	-
MP-CH-40	ROE DEER	blood	Haute-Garonne (31)	NA	A
MP-CH-41	ROE DEER	blood	Haute-Garonne (31)	NA	-
MP-CH-58	ROE DEER	blood	Haute-Garonne (31)	2	A
MP-CH-61	ROE DEER	blood	Haute-Garonne (31)	NA	-
MP-CH-63	ROE DEER	blood	Haute-Garonne (31)	-	-
MP-CH-64	ROE DEER	blood	Haute-Garonne (31)	2	-
MP-CH-65	ROE DEER	blood	Haute-Garonne (31)	2	A
MP-CH-66	ROE DEER	blood	Haute-Garonne (31)	NA	-
MP-CH-68	ROE DEER	blood	Haute-Garonne (31)	NA	-
MP-CH-69	ROE DEER	blood	Haute-Garonne (31)	NA	-
MP-CH-70	ROE DEER	blood	Haute-Garonne (31)	NA	-
NO-BO-01	CATTLE	blood	Calvados (14)	4	B
NO-BO-02	CATTLE	blood	Calvados (14)	4	B
NO-BO-03	CATTLE	blood	Pas-de-Calais (62)	4	B
NO-BO-04	CATTLE	blood	Calvados (14)	1	C
NO-BO-05	CATTLE	blood	Calvados (14)	1	C
NO-BO-06	CATTLE	blood	Calvados (14)	4	B
NO-BO-07	CATTLE	blood	Calvados (14)	4	B
NO-BO-09	CATTLE	blood	Calvados (14)	1	-
NO-BO-10	CATTLE	blood	Calvados (14)	NA	-
NO-BO-12	CATTLE	placenta	Calvados (14)	NA	-
NO-BO-14	CATTLE	mucus	Calvados (14)	NA	-
NO-BO-15	CATTLE	placenta	Calvados (14)	1	-
NO-BO-16	CATTLE	placenta	Calvados (14)	-	-
NO-BO-18	CATTLE	placenta	Calvados (14)	4	-
NO-BO-19	CATTLE	placenta	Calvados (14)	1	B
NO-BO-20	CATTLE	placenta	Calvados (14)	1	-

NO-BO-21	CATTLE	blood	Calvados (14)	NA	-
NO-BO-22	CATTLE	blood	Manche (50)	1	C
NO-BO-23	CATTLE	blood	Calvados (14)	NA	-
NO-BO-24	CATTLE	blood	Calvados (14)	4	B
NO-BO-25	CATTLE	blood	Calvados (14)	4	B
NO-BO-26	CATTLE	blood	Calvados (14)	1	B
NO-BO-27	CATTLE	blood	Calvados (14)	4	B
PL-BO-01	CATTLE	blood	Mayenne (53)	4	B
PL-BO-02	CATTLE	blood	Mayenne (53)	4	B
PL-BO-03	CATTLE	blood	Mayenne (53)	1	C
RA-BO-01	CATTLE	blood	Haute-Savoie (74)	4	B
RA-BO-02	CATTLE	blood	Rhône (69)	NA	-
RA-BO-03	CATTLE	blood	Rhône (69)	4	C
RA-BO-04	CATTLE	blood	Rhône (69)	1	-
RA-BO-05	CATTLE	blood	Rhône (69)	1	C
RA-BO-08	CATTLE	blood	Loire (42)	4	B
RA-DO-01	DOG	blood	Rhône (69)	NA	C
RA-DO-02	DOG	blood	Saône-et-Loire (71)	1	C
RA-DO-03	DOG	blood	Rhône (69)	1	C
RA-HO-02	HORSE	blood	Doubs (25)	1	C
RA-HO-03	HORSE	blood	Rhône (69)	1	C
RA-HO-04	HORSE	blood	Seine-et-Marne (77)	1	C
RA-HO-05	HORSE	blood	Saône-et-Loire (71)	1	C
CALL2	ROE DEER	blood	Côtes-d’Armor (22)	NA	-
PLO2	ROE DEER	blood	Côtes-d’Armor (22)	NA	-
PLU	ROE DEER	blood	Côtes-d’Armor (22)	NA	-
