# Supplementary material for: Multilocus sequence analysis of Anaplasma phagocytophilum reveals three distinct lineages with different host ranges in clinically ill French cattle
Source: Vet Res. 2014 Dec 9;45:114. doi: 10.1186/s13567-014-0114-7 (PMC4334609; doi:10.1186/s13567-014-0114-7)
Supplement: Additional file 2: — Primers used in and information about the nested PCRs. The table contains information on the primers used in the study. * developed by Lotrič-Furlan [30]. [file 13567_2014_114_MOESM2_ESM.pdf]

**Locus**	**Primers**	**Sequence en 3'→ 5'**	**Annealing temperature**
ankA external	Fi_ankA<br>Ri_ankA	ATRTTACGCTGTRRTRGCAT<br>CATCTRCHGGTGTTYTTCCCTC	58 °C
ankA internal	Fe_ankA<br>Re2_ankA	TATGCGRTTCGTGAAGCA<br>TTAAAAAGCATYTTTCTGWAAC	58 °C
msp4 external	msp4_Fb1<br>msp4_Rb1	ATAATGATGCGTCTGATGTTAGCG<br>CCTTTCCTCTATAAGCCAACTT	59 °C
msp4 internal	msp4_Fb1<br>msp4_Rb2	ATAATGATGCGTCTGATGTTAGCG<br>CAGGCAAGTCAGTATGCATAAC	59 °C
groEL external *	HS1a<br>HS6a	AZTGGGCTGGTAZTGAAAT<br>CCZCCZGGZACZAZACCTTC	48 °C
groEL internal *	HS43<br>HSVR	ATWGCWAARGAAGCATAGTC<br>CTCAACAGCAGCTCTAGTAGC	48 °C
polA external	F1_pola_deg<br>R3_pola_deg	GCAGATTAYTCVCARATGGARC<br>GCVGTACCYTGKATTGGWG	57 °C
polA internal	F1_pola_deg<br>R4_pola_deg	GCAGATTAYTCVCARATGGARC<br>GTACCYTGKATTGGWGCRTT	57 °C
typA external	E2_typA_F<br>E2_typA_R	CCTGGACATGCTGACTTCGG<br>CGGCGGAACTAACCTCACAG	66 °C
typA internal	F-typA<br>R-typA	TGCCTCTGAGGGCCCTATGCC<br>AGCCCTTTCCAGCCCTGCAAC	69 °C
recG external	recG_degF1<br>recG_degR2	CGRCTTGTAGTYATAGATG<br>TCRATGACAATKATSGTWGC	57 °C
recG internal	recG_degF2<br>recG_degR2	GATGARCARCARMGSTTYGG<br>TCRATGACAATKATSGTWGC	57 °C
pleD external	E2_pleD_R<br>R_pleD	ACAAGTGGCCCTGAAGCAAT<br>TGCGTCGTAGCCTGTCTGCA	66 °C
gyrA external	Fint-gyrA<br>Rext-gyrA	GATAGTCAGGGAAACTTCGG<br>GGCATGACACTAGTAATGGTC	61 °C
gyrA internal	Fint-gyrA<br>Rint-gyrA	GATAGTCAGGGAAACTTCGG<br>CATGTACGCTCCCTAAGCG	64 °C
CtrA-APH_1100 external	E1_aph1099-1100_F<br>E1_aph1099-1100_R	ACAGTGCCCAACCTAGACGA<br>TGGAAGAACACGGTGGTTGC	64 °C
CtrA-APH_1100 internal	f_aph1099-1100<br>r_aph1099-1100	GTTGCACATCCTGCTGGGGTGT<br>GCCCCTCTGCAGACAAAGAAGC	69 °C
