# Supplementary figures and images for: Multilocus sequence analysis of Anaplasma phagocytophilum reveals three distinct lineages with different host ranges in clinically ill French cattle
Source: Vet Res. 2014 Dec 9;45:114. doi: 10.1186/s13567-014-0114-7 (PMC4334609; doi:10.1186/s13567-014-0114-7)

d)

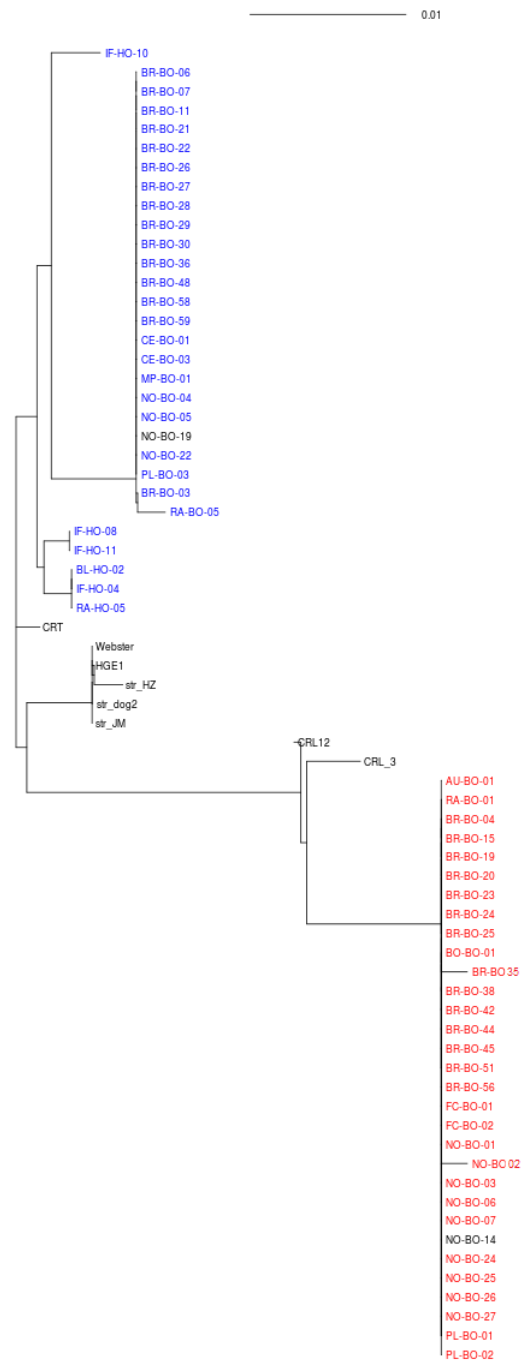

e)

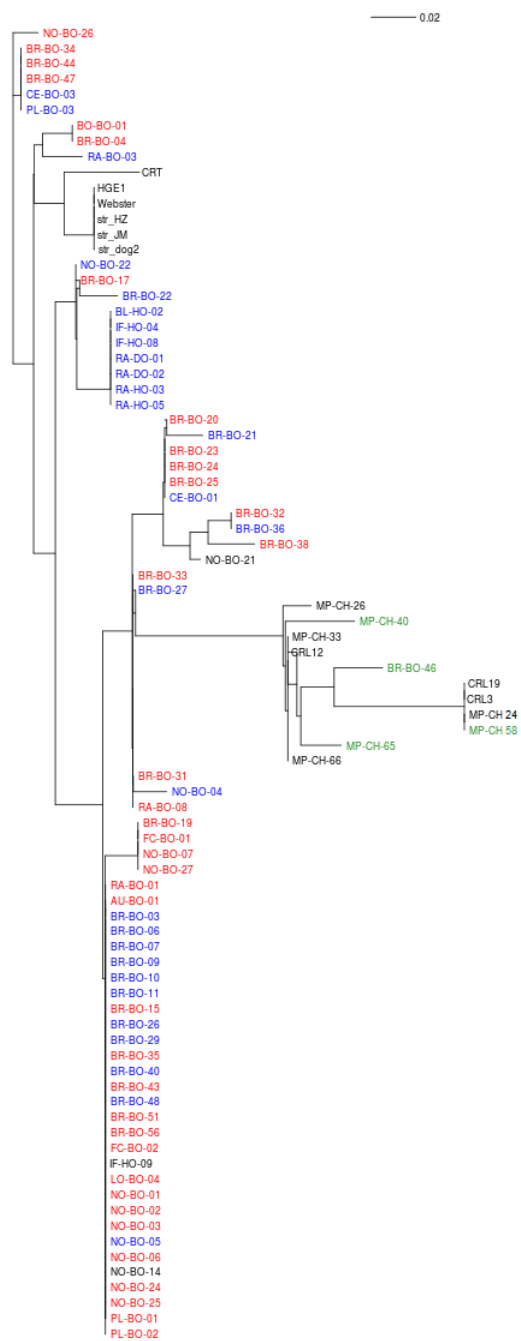

f)

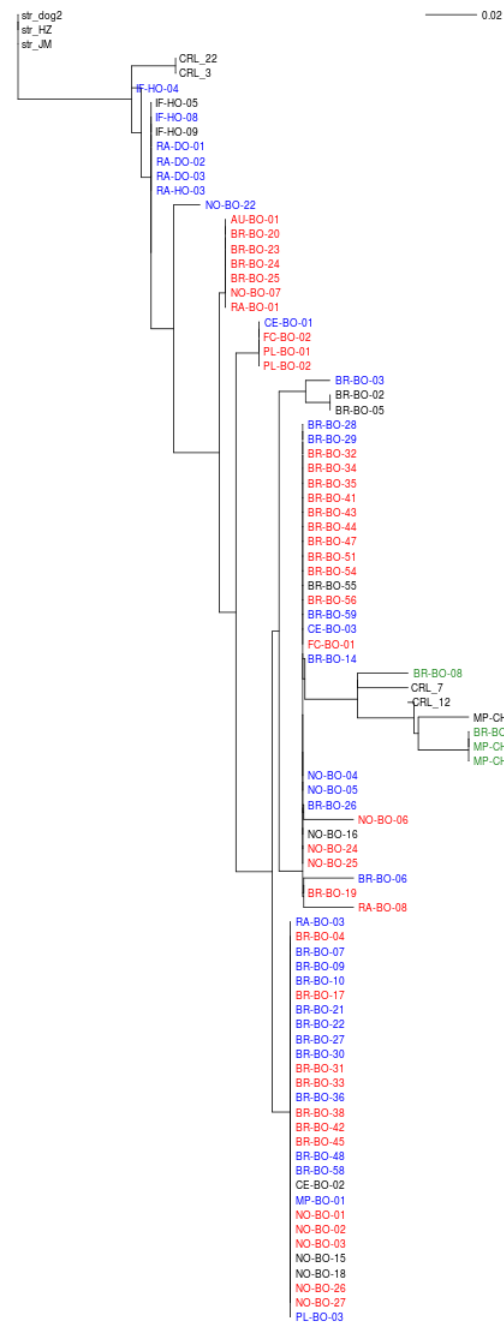

g)

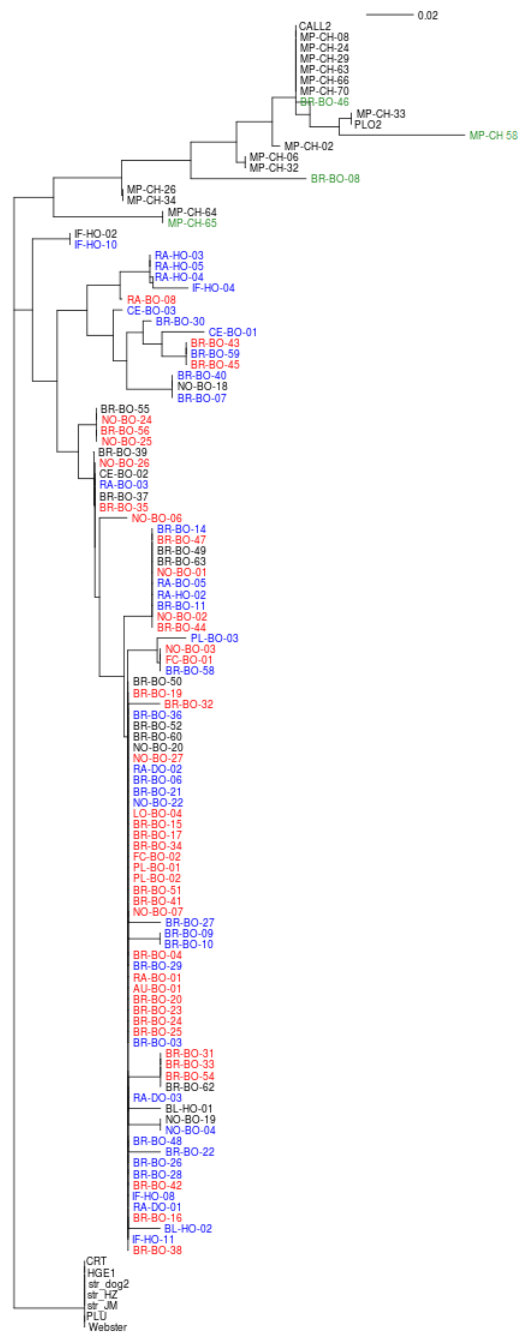

h)

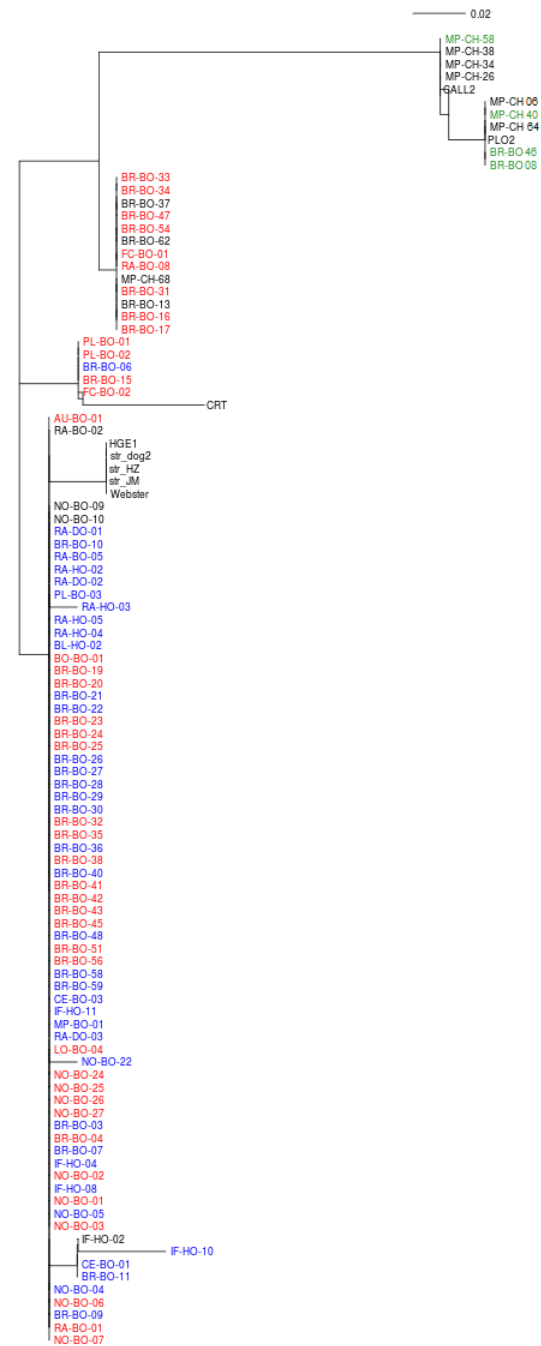

Supplement: Additional file 3: — Unrooted trees based on the sequences of the following loci: a) groEL; b) msp4; c) gyrA ; d) pleD ; e) polA ; f) recG ; g) typA ; and h) the intergenic region C trA- APH_1100. Each tree was built using a maximum likelihood approach; parameter optimization was performed with phymltest in R. The colors indicate the supertree cluster to which the samples were assigned: green for the cluster A, red for the cluster B, and blue for the cluster C. [file 13567_2014_114_MOESM3_ESM.pdf]

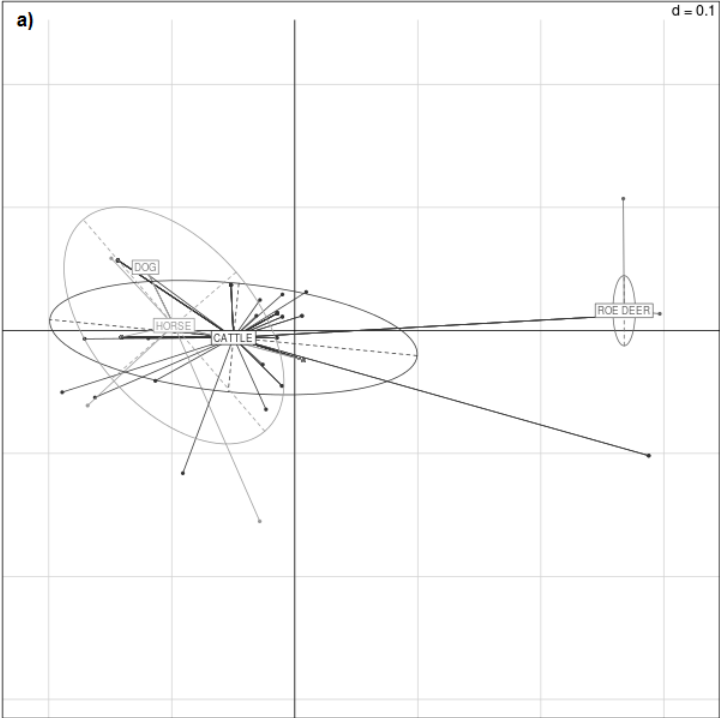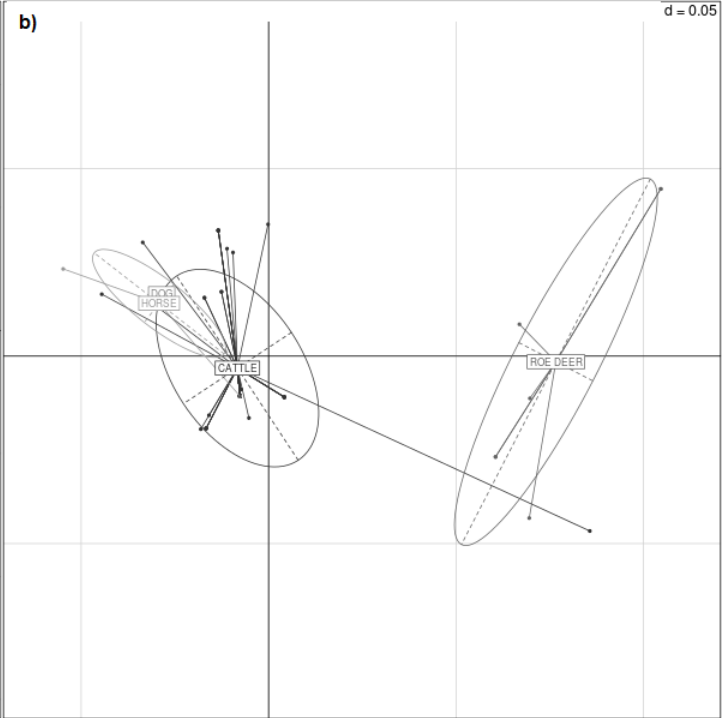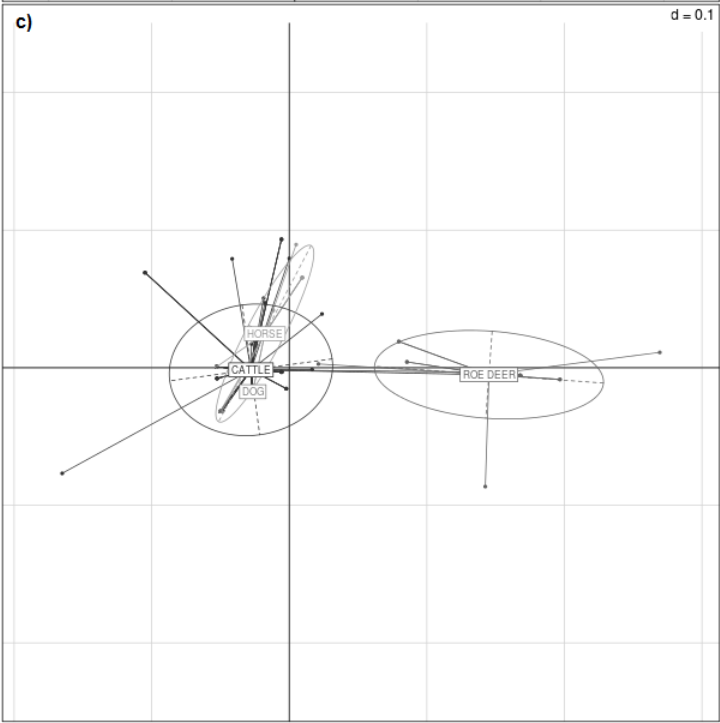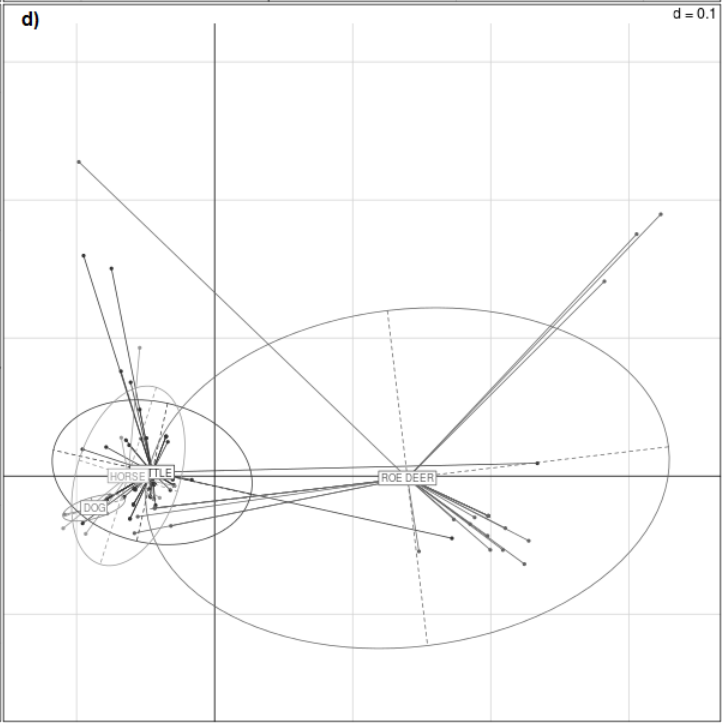

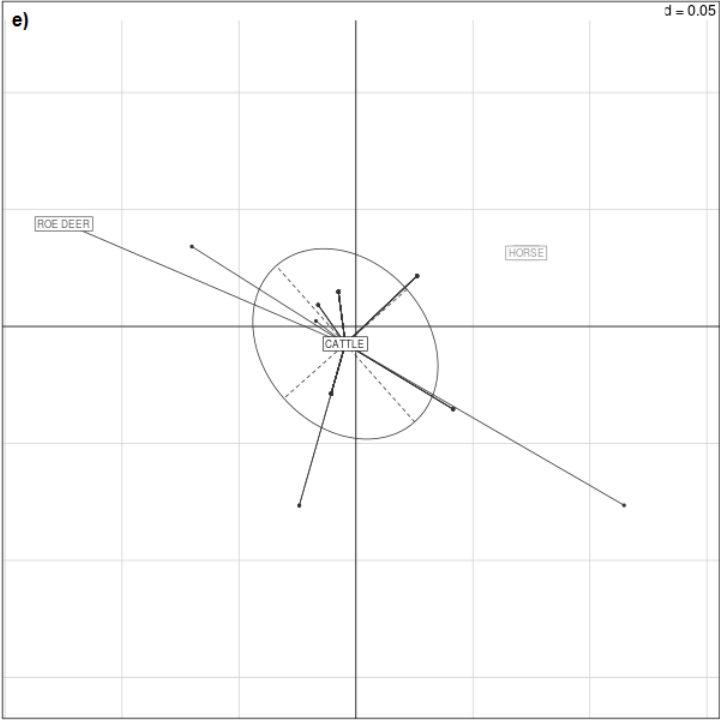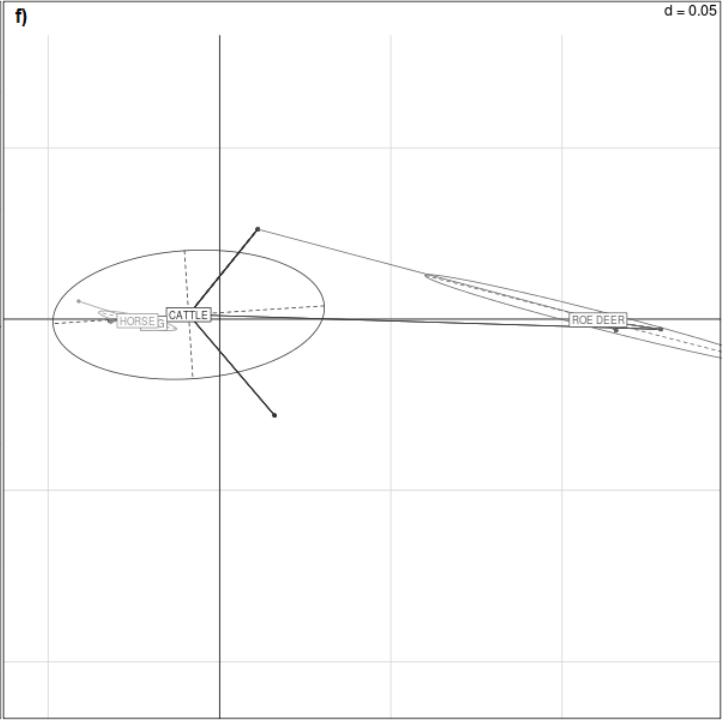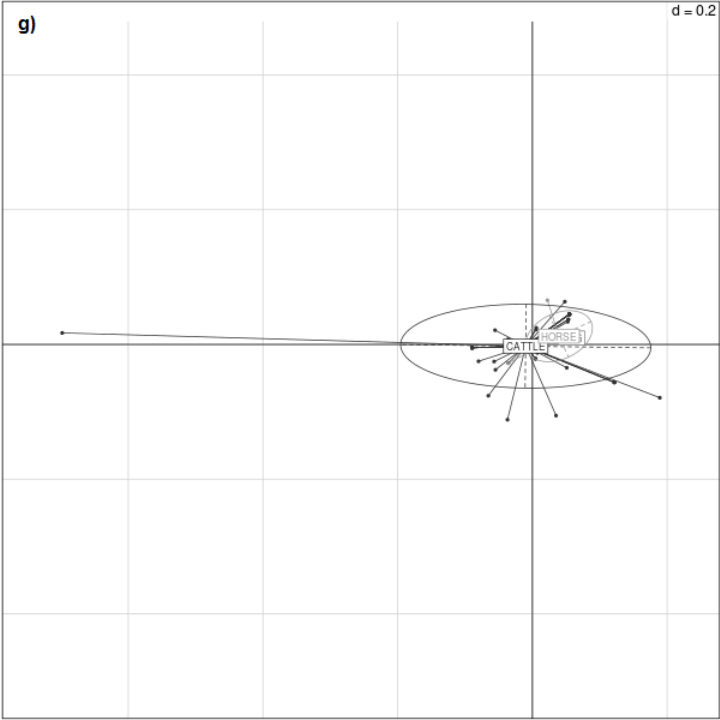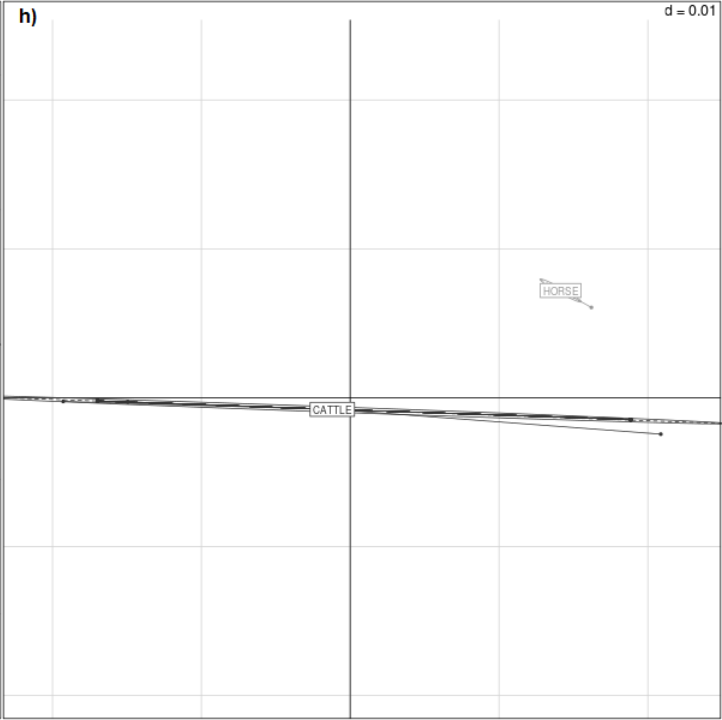

Supplement: Additional file 4: — Discriminant analysis by host based on the principal coordinates analyses of the following loci: a) msp4 ; b) polA ; c) typA ; d) groEL ; e) recG ; f) CtrA- APH_1100 ; g) gyrA ; and h) pleD. The graphs show the results of the discriminant analysis conducted on the principal coordinates analysis of each locus. [file 13567_2014_114_MOESM4_ESM.pdf]
